# Evolutionary programming as a platform for *in silico *metabolic engineering

**DOI:** 10.1186/1471-2105-6-308

**Published:** 2005-12-23

**Authors:** Kiran Raosaheb Patil, Isabel Rocha, Jochen Förster, Jens Nielsen

**Affiliations:** 1 Center for Microbial Biotechnology, BioCentrum-DTU, Building 223, Technical University of Denmark, DK-2800 Kgs. Lyngby, Denmark; 2 Centro de Engenharia Biológica, Universidade do Minho, 4710-057 Braga, Portugal; 3 Fluxome Sciences A/S, Søltofts Plads, Building 223, DK-2800 Kgs. Lyngby, Denmark

## Abstract

**Background:**

Through genetic engineering it is possible to introduce targeted genetic changes and hereby engineer the metabolism of microbial cells with the objective to obtain desirable phenotypes. However, owing to the complexity of metabolic networks, both in terms of structure and regulation, it is often difficult to predict the effects of genetic modifications on the resulting phenotype. Recently genome-scale metabolic models have been compiled for several different microorganisms where structural and stoichiometric complexity is inherently accounted for. New algorithms are being developed by using genome-scale metabolic models that enable identification of gene knockout strategies for obtaining improved phenotypes. However, the problem of finding optimal gene deletion strategy is combinatorial and consequently the computational time increases exponentially with the size of the problem, and it is therefore interesting to develop new faster algorithms.

**Results:**

In this study we report an evolutionary programming based method to rapidly identify gene deletion strategies for optimization of a desired phenotypic objective function. We illustrate the proposed method for two important design parameters in industrial fermentations, one linear and other non-linear, by using a genome-scale model of the yeast *Saccharomyces cerevisiae*. Potential metabolic engineering targets for improved production of succinic acid, glycerol and vanillin are identified and underlying flux changes for the predicted mutants are discussed.

**Conclusion:**

We show that evolutionary programming enables solving large gene knockout problems in relatively short computational time. The proposed algorithm also allows the optimization of non-linear objective functions or incorporation of non-linear constraints and additionally provides a family of close to optimal solutions. The identified metabolic engineering strategies suggest that non-intuitive genetic modifications span several different pathways and may be necessary for solving challenging metabolic engineering problems.

## Background

Microorganisms are widely used for producing antibiotics, therapeutic proteins, food and feed ingredients, fuels, vitamins and other chemicals. Currently there is an increasing trend to replace chemical synthesis processes with biotechnological routes based on microbial fermentations. In order to economically produce desired compounds from microbial cell factories it is, however, generally necessary to retrofit the metabolism, since microorganisms are typically evolved for maximizing growth in their natural habitat. Retrofitting of microbial metabolism has traditionally been done through classical strain improvement that involved random mutagenesis and screening, whereas in later years rational design strategies based on genetic engineering have been applied with an increasing success – often referred to as metabolic engineering. In metabolic engineering many experimental and mathematical tools have been developed for introducing directed genetic modifications that will lead to desirable metabolic phenotypes resulting in improved production of desirable compounds or in reduced production of by-products [1,2]. Until now most of the successes in metabolic engineering have been based on qualitative or intuitive design principles. However, even though there are several success stories in metabolic engineering there are also many attempts that have failed due to the lack of rational strategies based on predictive analysis tools.

Microbial metabolism is often subjected to tight regulation and is constrained by mass and energy conservation laws on a large number of intracellular metabolites, and this makes it difficult to predict the effects of introducing genetic modifications in a given cell. Moreover, as metabolic pathways and related regulatory processes form complex molecular and functional interaction networks [3,4], it is only through analysis of the metabolism as a whole in an integrative systems approach [5] that one may evaluate the effect of specific genetic modifications. Genome-scale models of microbial organisms [6], comprising different levels of information, primarily on the stoichiometry of the many different reactions but possibly also comprising some information about regulation, could offer a suitable platform for developing systems level tools for analyzing and engineering metabolism [7]. Although there have been some attempts to simulate dynamic behavior of whole cell systems [8,9], currently these approaches enjoy limited applicability due to lack of kinetic and regulatory information on the whole genome scale. Nevertheless, in absence of kinetic and regulatory information it is possible to at least partly predict the behavior of cellular metabolism by using steady state analysis based on genome-scale stoichiometric models.

Genome-scale stoichiometric models represent the integrated metabolic potential of a microorganism by defining flux-balance constraints that characterizes all feasible metabolic phenotypes under steady state conditions. Because of the large number of reactions occurring in cellular metabolism, the dimensions of the solution space (or the number of feasible metabolic phenotypes) defined by genome-scale models [10,11] is very large. Consequently, combinatorial complexity prevents calculation of all feasible metabolic phenotypes that a microbial genotype can assume under a given environmental conditions [12]. One of the approaches to determine the metabolic phenotype (i.e. the fluxes through all metabolic reactions) is to use flux balance analysis (FBA) [13,14]. In FBA a particular flux or a linear combination of various fluxes (objective function) in the model is optimized through linear programming, thus leading to a solution to the fluxes through all metabolic reactions. Since several microbial metabolic networks have evolved towards operation of optimal growth rate [15-18], the use optimization of growth rate is an often applied objective function in FBA. There are, however, some other approaches to determine flux distributions, especially for deletion mutants that might not be capable of realizing the same objective function as the wild-type strain [19-21]. Nevertheless, all these methods provide a basis for using genome-scale metabolic models to predict possible metabolic phenotypes, and hence for *in silico *metabolic engineering. However, despite of their potential, genome-scale stoichiometric models have been scarcely used for metabolic engineering purposes.

The algorithm developed by Maranas *et al*. [22,23] (named OptKnock) represents one of the first rational modeling frameworks for suggesting gene knockouts leading to the overproduction of a desired metabolite. OptKnock searches for a set of gene (reaction) deletions that maximizes the flux towards a desired product, while the internal flux distribution is still operated such that growth (or another biological objective) is optimized. Thus the identified gene deletions will force the microorganism to produce the desired product in order to achieve maximal growth. Indeed, the design philosophy underlying OptKnock approach takes advantage of inherent properties of microbial metabolism to drive the optimization of the desired metabolic phenotype. The relation of OptKnock with the biological objectives of microorganisms makes it an attractive and promising modeling framework for *in silico *metabolic engineering.

OptKnock is implemented by formulating a bi-level linear optimization problem using mixed integer linear programming (MILP) [22] that guarantees to find the global optimal solution. In this report, we extend the applicability of OptKnock approach by formulating the *in silico *design problem by using a Genetic Algorithm (GA), hereafter referred to as OptGene. Genetic algorithms use the principle of Darwinian evolution to search (*evolve *through mutations and reproduction) for the global optimal solution (individual with a maximum *fitness *score). Direct relation of GA with biological evolution makes it a natural method of choice to identify suitable genetic modifications for improved metabolic phenotype. There are two major advantages of the OptGene formulation. Firstly, OptGene demands relatively less computational time and thus it enables to solve problems of larger size. This is of particular importance as the relation between the size of the problem (as defined by the number of enzymes and number of deletions desired) and the corresponding search space (combinations of enzymes to be deleted) is combinatorial (Supplementary Figure 1) [see Additional file 1]. Thus, the number of possible combinations of 5 reaction-deletions in a model with 250 reactions is more than 7.8 × 10^9^, whereas existing genome-scale stoichiometric models comprise a significantly higher number of reactions. Secondly, the OptGene formulation allows the optimization of non-linear objective functions, which is of considerable interest in several problems of commercial interest. One example of an important non-linear engineering objective function is the productivity (amount of product formed per unit time).

**Figure 1:**
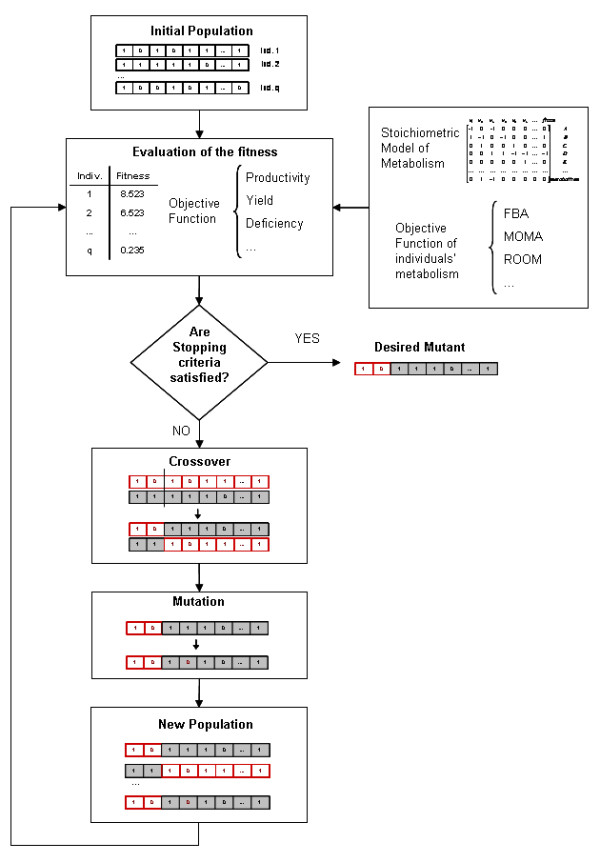
**Schematic overview of the OptGene algorithm. **A population of individuals is initiated by specifying a present/absent status for each gene in each of the individuals. Individuals are then scored for their fitness by using FBA/MOMA/other method of choice and the objective function (/s). Individuals are selected for mating based on their fitness score, and subsequently crossed to produce new offspring. Mutations are introduced in individuals randomly at specified mutation rate and thus a new population is obtained. This cycle of evolution is repeated until a mutant (or mutants) with a desired phenotypic characteristics is obtained. Please refer to the text for detailed description of each step in the algorithm. Grey shaded or red walled boxes are used to represent different individuals in the cross-over process. Ind.- Individual. FBA- Flux balance analysis [13,14]. MOMA- Minimization of the metabolic adjustment [19]. ROOM- Regulatory on/off minimization [20].

## Results and Discussion

### OptGene algorithm

Two different versions of the OptGene algorithm were used in this work, differing mainly on the representation of the metabolic genotype: binary (binOptGene) and integer (intOptGene) representations. The binary form of the OptGene algorithm is schematically illustrated in Figure 1, and the important steps of both representations are explained in the following.

#### Model pre-processing

Since GA do not exhaustively search the complete solution space, it is necessary to avoid local optimal solutions by proper formulation of the problem. We therefore pre-processed the model to remove duplicate and dead-end reactions. Also a linear pathway (or enzyme subset [24]) was represented as a single reaction in GA. Moreover, lethal reactions (including genes that are found to be lethal *in vivo*, but not *in silico*) were not included as the possible targets in GA. This pre-processing step reduced the problem size considerably and thus reduced the number of local optimal solutions (data not shown).

#### Chromosome representation of metabolic genotype

**Figure 2:**
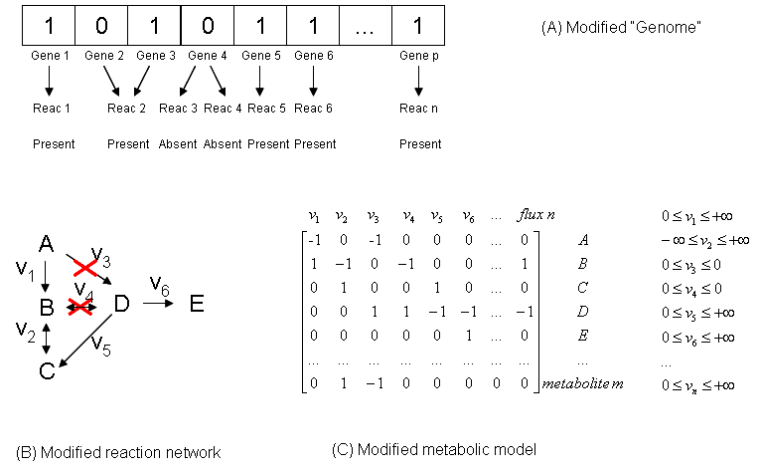
**Representation of the metabolic genotype. **Each gene of the microorganism is assigned a binary value, representing its absence/presence in the mutant (A). The individual genes are associated with one or more reactions in the metabolic network (B). When a given reaction is in the absent status, the upper and lower bonds for the corresponding metabolic flux are set to zero, resulting in a modified metabolic model (C).

Each reaction in the metabolic model can be associated with one or more genes in the genome. In the binOptGene algorithm each of those genes is represented by a binary variable indicating its absence/presence (0/1), and thus a set of these variables forms an "individual" (sometimes also referred to as "chromosome" in evolutionary algorithms nomenclature) representing a particular mutant that lacks some metabolic reactions when compared with the wild type (Figure 2). For the intOptGene implementation, the individuals are composed of integer numbers representing only the genes to be deleted, according to their relative order in the metabolic model. This way, the number of genes to be deleted can be directly imposed by changing the size of the individuals. The phenotypes of every individual can be obtained by using FBA or other algorithms. The problem then is to find the set of genes to be deleted from an individual so as to obtain a desired phenotype (e.g. with maximum product yield and minimum undesired by-product yield).

#### Initialization of population

The GA begins with a predefined number of individuals, forming a population. In the binOptGene, individuals in the population can be initialized in different ways, e.g. by assigning present/absent status to each gene randomly, or assigning present status to all genes, while in the intOptGene representation, the population is usually initialized randomly.

#### Scoring fitness of individuals

Each individual is assigned a fitness score that determines whether it will reproduce and/or propagate to the next generation. The fitness score of an individual is calculated using the desired objective function value. The objective function value can be calculated using FBA, minimization of metabolic adjustment (MOMA) [19], regulatory on-off minimization (ROOM) [20] or any other algorithm. The GA by itself is independent of scoring algorithm.

#### Crossover of chromosomes

After the fitness score is calculated for all individuals in the population, the best individuals are selected for crossover. A selection scheme that is most commonly used is the Roulette wheel, where individuals are selected based on the magnitude of the fitness score relative to the rest of the population. The higher the score, more likely an individual will be selected. Selected individuals are then crossed to produce a new offspring. The crossover methods used in this study were one-point, two-points, and uniform crossover [25].

#### Mutation

Individuals propagating to the new population are mutated (in our formulation, a gene is deleted) with a given probability.

#### New population and termination

Mutation and crossover give rise to a new population, which can then again be subjected to a new round of evaluation, crossover and mutations. This cycle is repeated until an individual with a satisfactory phenotype is found.

We illustrate the principles and utility of OptGene algorithm by using three interesting metabolic engineering problems with the yeast *Saccharomyces cerevisiae*, which is one of the most widely used cell-factories. We applied OptGene for *S. cerevisiae *to identify gene-deletion strategies for improving yield and substrate-specific productivity of three metabolites, namely vanillin, glycerol and succinate. The yield of a product (metabolite) of interest is defined as the grams of product produced per unit gram of the substrate consumed, whereas substrate-specific productivity is defined as the grams of product produced per unit time per unit substrate consumed. It is important to note that models based only on stoichiometry can not predict rates without an assumption of a fixed substrate uptake rate. Since the substrate uptake rates for deletion mutants might change substantially and the fact that it is very difficult to predict such changes *a priori*, in general the productivity can not be optimized by using stoichiometric models. One of the ways to circumvent this problem is to optimize the function [Product Yield × Growth]. Although, this quantity will be equal to the substrate-specific productivity under the assumption of a fixed substrate uptake rate, we will refer to this term as Biomass-Product Coupled Yield (BPCY) rather than the productivity as this may cause confusion (also see Note 1 for comments about the growth rates for mutants). BPCY represents an interesting example of a non-linear objective function that can be optimized by using OptGene.

### Vanillin case study

Vanillin is a natural flavor compound extracted from plants and is widely used as a food ingredient. There is some commercial interest in producing vanillin by using recombinant microorganisms and in particular *Saccharomyces cerevisiae *which is a food grade organism. Since vanillin is not produced naturally by *S. cerevisiae*, the corresponding reactions were inserted into the model as suggested by Pharkya *et al*[23]. Then we used OptGene to find gene deletion strategies to improve the BPCY as well as the yield of vanillin. We found that it was possible to improve the vanillin yield *in silico *up to 90 % of the theoretical limit by deleting only 2 reactions (pyruvate decarboxylase and glutamate dehydrogenase), while keeping the growth rate at 60% of the parental strain. A similar strategy was predicted for a mutant with the maximum BPCY. The suggested strategy diverts the pyruvate flux going to ethanol towards vanillin where NADH is oxidised back to NAD^+^. Furthermore, deletion of glutamate dehydrogenase results in an increased availability of NADPH needed for vanillin biosynthesis. Increasing the allowable number of deletions did not result in substantial improvement in the yield or BPCY.

### Glycerol case study

Currently glycerol is mainly recovered as a by-product from soap manufacturing or produced from propylene and is widely used to synthesize several products ranging from cosmetics to lubricants [26]. Alternatively, glycerol can also be produced through microbial fermentation using sustainable carbohydrate resources. *Saccharomyces cerevisiae *naturally produces glycerol in small quantities during anaerobic fermentation or under osmotic stress. Moreover, glycerol plays an important role in maintaining the cytosolic redox balance under anaerobic conditions and it is therefore interesting to study the effects of gene-deletions on yield and productivity of glycerol. We applied the OptGene algorithm to identify gene deletions leading to improved yield and BPCY of glycerol under aerobic conditions, where the maximum theoretical yield of glycerol is much higher as opposed to anaerobic fermentation.

**Figure 3:**
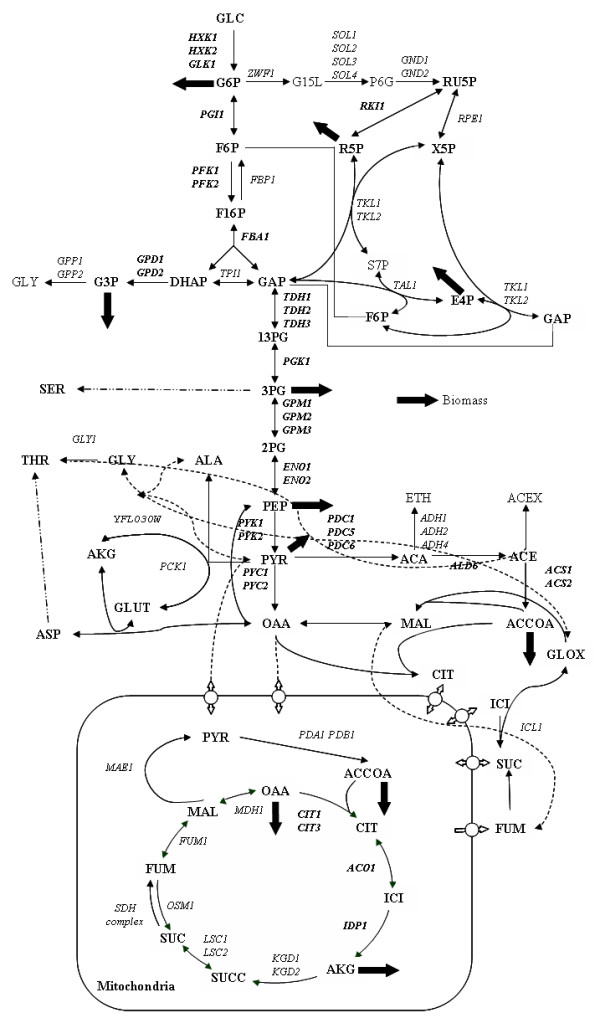
**Schematic overview of the *Saccharomyces cerevisiae *central carbon metabolism. **The figure shows important pathways in the central carbon metabolism including certain branch points towards the amino acid metabolism. The thick arrows indicate the drain of metabolites towards biomass production. Arrows with the  style indicates a lumped pathway. Multiple names for a reaction indicate the presence of iso-enzymes. The nomenclature of the metabolites can be found in the Supplementary table 1 [see Additional file 1]. The figure is partially adapted from Forster et al. (2002) [32].

Results suggested that no single gene deletion will result in glycerol production, whereas a strategy for double reaction deletion is identified, namely FBP1 (Fructose-1,6-bisphosphatase) and genes encoding Glyceraldehyde-3-phosphate dehydrogenase (*TDH1*, *TDH2 *and *TDH3*). This strategy makes intuitive sense as reactions that branch the flux away from dihydroxyacetone phosphate (the precursor for glycerol) are deleted (see figure 3 for a schematic representation of yeast central carbon metabolism). With this strategy it is possible to obtain a yield of 0.49 g/g-glucose with a corresponding growth rate that is 80% lower than the reference strain. Increasing the number of deletions up to six did not result in a further substantial increase in the yield. However, interestingly, the BPCY of glycerol improved with the number of deletions allowed. With six deletions, the BPCY reached up to 41 mg/g glucose-hr (yield of 0.31 g/g-glucose) with a growth rate equal to 50% of that of the reference strain. Moreover, the identified deletions for yield and BPCY improvement are different (Supplementary Table 2) [see Additional file 1]. Notably, the suggested deletions span not only the central carbon metabolism but also extend to amino acid and vitamin metabolism, illustrating the tight links between different metabolic pathways arising from the mass balance constraints. This also illustrates the need for the here reported algorithm which can search this vast solution space efficiently.

### Succinic acid case study

Succinic acid is one of the intermediates of the TCA cycle and is an interesting chemical to be used as a feedstock for synthesis of a wide range of chemicals. As a metabolite from the central carbon metabolism, it is a good case study for devising metabolic engineering strategies. Multiple gene deletion strategies obtained using OptGene algorithm for improving succinic acid yield and BPCY are summarized in table 1.

**Table 1:** Different deletion strategies suggested by OptGene algorithm for improving succinate yield and Biomass Product Coupled Yield.

**Objective function**	**Number of deletions**	**Suggested deletions^1^**	**Objective function value^2^**	**%Maximum Growth**	**Unique solution?^3^**
Succinate yield	5	*SDH-complex, ZWF1, PDC6, U133, U221*	0.39	14%	Yes
		*SDH-complex, ZWF1, PDC6, U133, U41*	0.37	1%	Yes
	4	*SDH-complex, ZWF1, PDC6, AGP3*	0.356	30%	Yes
	3	*SDH-complex, ZWF1, PFK2*	0.211	4%	Yes
		*SDH-complex, SER3, THR1*	0.074	76%	Yes
Succinate Biomass Product Coupled Yield	4	*SDH-complex, ZWF1, PDC6, AGP3*	29	30%	Yes
		*SDH-complex, SER3, THR1, U221*	22	75%	Yes
	3	*SDH-complex, SER3, THR1*	16	76%	Yes
		*SDH-complex, ZWF1, GLT1*	9.78	42%	Yes

^1 ^Only few of the suggested strategies, with high objective function values are shown. OptGene found many strategies with different, but high objective function values. This tendency can be controlled by varying GA parameters.

^2 ^Units are: Yield in gram (gram glucose)^-1^, Biomass Product Coupled Yield in milli-gram (gram-glucose.hour)^-1^

^3 ^Uniqueness of the solution was verified by first optimizing for the biomass, and then minimizing and maximizing the succinate flux at fixed, optimal biomass value.

Firstly, we note that the maximum theoretical yield of succinic acid is 0.506 g/g glucose (Note 2) when no biomass is being produced, and that no succinic acid can be produced at optimal biomass growth rate. Moreover, no single gene deletion strategy resulted in succinic acid production. For a double gene deletion strategy, deletion of the *SDH-complex *(succinate dehydrogenase) and *THR1 *(homoserine kinase) is predicted to result in a succinic acid yield of 0.018 g/g glucose, with a 10% reduction in the growth rate. Flux re-distribution leading to this improvement in the double-deletion mutant is quite interesting and non-intuitive. Deletion or inactivation of the *SDH-complex *prevents the conversion of mitochondrial succinate to fumarate, while simultaneous deletion of *THR1 *forces threonine synthesis via glycine, which may be formed from glyoxylate. Consequently there is increased flux through *ICL1 *(cytosolic isocitrate lyase, catalyzing the reaction from isocitrate to glyoxylate and succinate), thus creating surplus succinate that is secreted by the cell. Moreover, this flux re-distribution is also associated with an increased flux through the pentose phosphate (PP) pathway for increased NADPH availability. We note that in the mutant with only the *SDH-complex *deleted, threonine is synthesized via aspartate, which is optimal route for maximizing biomass production. The same double deletion mutant was also predicted to show maximum BPCY (4.5 mg/g glucose-hr).

The search for a triple deletion mutant with maximum succinate yield suggested deletion of the *SDH-complex*, *ZWF1 *(Glucose-6-phosphate dehydrogenase) and *PFK2 *(Phosphofructokinase). Although this resulted in increased prediction of succinate yield (0.21 g/g glucose), the corresponding growth rate is very low (96% reduction in growth rate), making this solution unattractive. However, a triple deletion mutant with maximum BPCY (16 mg/g glucose-hr) was found to have 76 % of the wild-type growth rate and a succinate yield of 0.07 g/g glucose. The corresponding solution suggested deletion of *SER3 *in addition to the double deletion strategy discussed above. Deletion of *SER3 *blocks the synthesis of L-Serine via 3-Phospho-D-glycerate, which increases the demand on glycine production via glyoxylate. Overall, it leads to a further increase in the flux through *ICL1 *ensuring a higher flux towards succinate while maintaining a high growth rate. This increase is also associated with a further increase in the flux through the PP pathway.

In spite of a slow growing triple deletion mutant for improved yield, the algorithm found a quadruple deletion mutant with not only improved yield (0.36 g/ g glucose), but also with much higher growth rate, as compared to the triple deletion mutant (table 1), and therefore higher BPCY. The suggested genes for deletion are the *SDH-complex*, *ZWF1*, *PDC6 *(pyruvate decarboxylase) and *AGP3 *(glutamate permease). Deletion of *ZWF1 *increases the flux through glycolysis and deletion of *PDC6 *increases conversion of pyruvate to oxaloacetic acid via *PYC1*. This flux could be directed towards glutamate production and into the TCA cycle. But since the *SDH-complex *is deleted the flux through TCA cycle is limited, while deletion of secretion reaction for surplus glutamate forces malate formation from oxaloacetic acid. The flux through malate is then directed to succinate via fumarate. We also searched for a quadruple deletion strategy for maximum BPCY and the algorithm suggested the same deletion strategy as for the maximum yield, with a corresponding BPCY of 29 mg/g glucose-hr. This BPCY shows a substantial increase over the BPCY obtained with the triple deletion strategy.

Results of a further search allowing more gene deletions, for improvement in yield and BPCY, are summarized in table 1. Here we note that it might be difficult to realize some of the suggested optimal strategies *in vivo *due to a variety of reasons, e.g. regulatory constraints, orphan reactions etc. However OptGene provides not only the optimal solution found, but also generates a family of "good" solutions and thus provides many strategies that can be further analyzed manually before experimental verification. Some of such alternative solutions are also reported in table 1.

### MOMA approach

The examples discussed above use FBA as scoring function to evaluate fitness of an individual in the GA. However, as noted before, the flux distribution of mutants of *Echerichia coli *have been shown to be better approximated by assuming that the fluxes tend to have a minimum distance from wild-type flux distribution, which may not correspond to the flux distribution for maximum growth [19]. Nevertheless, although this approach, referred to as Minimization of Metabolic Adjustments (MOMA), might explain the flux distribution of mutants better than FBA, such mutants might approach towards FBA-predicted optimal solution when evolved under growth pressure [15,27].

To check whether the two approaches for evaluating flux distributions (namely FBA and MOMA) result in different predictions for multiple deletion mutants, we used OptGene to search for double and triple deletion mutants with improved succinic acid yield and BPCY. The double deletion strategy for obtaining maximum yield with MOMA includes deletion of *FUM1 *(fumarase) and *PDA1 *(pyruvate dehydrogenase). This strategy is different from that suggested by using FBA, and it also predicts a better yield (0.11 g/g glucose) for a double deletion mutant. In case of BPCY the MOMA approach yielded the same productivity, although with different genes (*RPE1 *and an orphan reaction in mitochondria). However, an effective comparison of FBA and MOMA for multiple deletion mutants can only be done after experimental evaluation.

### Significance and effects of different GA parameters

Parameterization of stochastic optimization methods like evolutionary algorithms is recognized as a difficult task and for this particular problem only an empirical study of the effect of different parameters was conducted. The main purpose of this parameterization was to be able to obtain a global optimum within a reasonable computation time.

Different sizes of the population were tested, and it was found that an increase beyond 125 individuals did not improve the results significantly. Furthermore, a mutation rate of 1/(genome size) was found to be optimal for both representations (Supplementary Figure 2) [see Additional file 1].

Regarding crossover methods, for the binOptGene representation, one-point, two-points and uniform crossover methods were tested, and the different crossover techniques gave almost the same results, indicating that all approaches are equally good, probably due to their similar operation mode. For intOptGene, only one type of crossover method was tested, namely uniform crossover where a child obtains a gene from each parent with equal probability.

**Figure 4:**
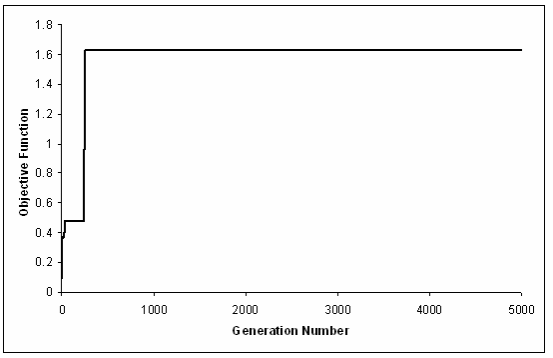
Typical shape of the convergence curve of OptGene.

After parameterization, for both representations, and for a typical optimization run, the evolutionary algorithms were able to achieve a solution within 1000 generation, although the algorithm was allowed to run until 5000 generations. A typical convergence curve can be found in Figure 4.

### Resemblance to Natural Evolution

The theoretical foundations of genetic algorithms rely on a notion of short, highly fit schemata, also known as building blocks (see e. g. [25,28]), that are propagated generation to generation and constitute the basis for the convergence to optimal solutions. For the strain design problem, building blocks can be regarded as subsets of genes in a close position on the individuals of the evolutionary algorithms that, when deleted together, improve process yield or productivity.

The differences on the representation of individuals in both approaches used in this work originate different requisites in terms of the formation of building blocks: as in the binary representation the order of the genes in the individuals follows closely that of the stoichiometric model (where genes are grouped according to the main pathways they integrate), only related genes can be a part of the building blocks. On the other hand, for the integer representation any subset of unrelated genes can form a building block. A natural conclusion of this observation is that the more the genes in the metabolic model follow a biological meaningful order, the more similar the binOptGene optimization approach is to a biological evolution of microorganisms under a given selective pressure.

Additionally, we observed that if the limitation on the number of genes to be deleted in binOptGene is imposed by using penalty functions after evaluation of individuals, the number of invalid individuals in the population at a given generation is very large and consequently negatively affects the convergence.

Nevertheless, in spite of the described differences, and although it is known that usually Genetic Algorithms do not perform very good for problems of the size found for the binary implementation, similar results were obtained for both approaches, after parameterization. In fact, for the majority of the runs, and with both representations, there was a clear convergence to an optimum (Figure 4), and the solutions found were very similar among all the repetitions (typical values of the relative standard deviation of 20 runs are 6%). Additionally, most of the times the final solution was found very early, indicating that 500–1000 generations are probably enough for converging to a satisfactory solution. However, by looking at the shape of the convergence curve in figure 4, it is clear that there are several sudden increases in the performance of the best individual, as opposed to the most often observed smooth convergence curves obtained with evolutionary algorithms. These step changes in the objective function value are usually an indication that the optimization is being stopped very prematurely but, as more iterations do not improve the final solution, it is more likely that the problem itself is discrete. In fact, and although additional characterization of the search space is needed, this observation can be explained by the evidence that, when a good candidate for deletion is found, the performance of the best individual in a population increases significantly.

### Global optimal solution and computational cost

In case of succinate yield optimization, the optimality of the solution found by OptGene was verified using exhaustive search with up to 4 deletions. In case of BPCY, although the optimal solution for 3 deletions represented a global optimum, for a 4 deletion case OptGene found a sub-optimal solution. However, this solution was quite close to the global optimal solution (85% of the global optimal value). With 5 deletions the optimal solution found reached quite close to the maximum possible BPCY value. We hereby note that in cases where global optimality can not be directly verified, a good estimate for closeness to the global optimal solution can be found by using a curve similar to that presented in Supplementary Figure 3 [see Additional file 1]. The plot in the Supplementary Figure 3 is generated by fixing the biomass yield at different values and then optimizing for the succinate production.

The computational cost of OptGene (estimated as the number of objective function evaluations necessary to find an optimal solution) was found to be 0.03 % of that found by using exhaustive search for 4 gene deletion case (succinate yield) and 0.33 % for succinate BPCY case. However, we did not observe any direct correlation between the number of deletions and the computational cost. Supplementary table 4 [see Additional file 1] summarizes the computational cost associated with the succinic acid optimization case.

### Multiple optima

Since the flux distribution obtained using FBA is not necessarily unique, the objective function value obtained in the fitness evaluation routine may not be unique as well. This is usually due to the possibility of other by-products being formed instead of the desired product (Supplementary Table 3 [see Additional file 1] lists the metabolites that were allowed to be secreted by the cells in this study). Consequently it has an important implication while designing the deletion strategies. Such check for uniqueness of objective function can easily be incorporated in the fitness evaluation routine by using flux variability analysis. Thus, e.g., an upper and lower bound can be calculated for the product flux at the optimal growth rate. The choice between "pessimistic" and "optimistic" fitness value can be left for the user. However, we note that for the results presented in this study, the solutions obtained were unique as indicated in the last column of Table 1.

## Conclusion

We report a GA based framework termed OptGene for designing microbial strains *in silico*. OptGene presents two major advantages, higher speed and ability to optimize for non-linear objective functions. The optimal solution for a four deletion problem (succinate yield case) was found using OptGene by searching only 0.03% of the total solution space. For a higher number of deletions, the OptGene search space represents considerably lower fraction of the total solution space that increases exponentially. As a consequence of an exponential increase in the search space, a detailed study of the correlation between the OptGene search space and the total solution space was not feasible. Nevertheless, as discussed in the results section, it is possible to estimate the closeness to the global optimal solution by comparing the results with the plots as reported in Supplementary Figure 3 [see Additional file 1]. Consequently, high computational speed of OptGene enables addressing the problems involving large number of genes, and searching for higher number of deletions. This is of particular interest as genome-scale models of simple eukaryotic organisms like *S. cerevisiae *include more than 1000 reactions. In case of simple minimal media that we used in our simulations, this set of 1000 reactions can be reduced to 240 reactions as described in the algorithm. This number can still be large for solving quadruple deletion problem using exhaustive search algorithms.

The metabolic engineering strategies reported in this work suggest that non-intuitive genetic modifications spanning several different pathways may be necessary for solving challenging metabolic engineering problems. Consequently *a priori *selection of candidate targets might lead to sub-optimal solution, and it is desirable to consider the whole model. Moreover, with the recent advances on the experimental front, it is feasible to construct mutants with many knockouts in real time. It should also be noted that we might often need to recalculate the results in case of changes/errors in the model, e.g. after including regulatory information or addition of a new reactions. Speed of calculations can be a key factor in such cases. OptGene can serve to provide a quick hint to whether a particular function of interest can be improved at all or up to what extent. The ability of OptGene to optimize for non-linear objective functions opens new opportunities for designing microbial strains with tailor-made metabolic phenotype, e.g. a strain with high BPCY of x and low yield of y.

The GA formulation can provide us with multiple solutions, and thus an opportunity to choose from many good solutions. This is of interest as many of the predicted solutions might be difficult to realize due to complex biological regulation, which is difficult to account for in scoring function models. Moreover, the GA framework is very flexible and thus can easily be changed to use different scoring functions depending on the problem and system under investigation. In conclusion, OptGene represents a computationally efficient, flexible and natural tool for *in silico *designing of microbial strains by using genome scale models.

## Methods

### Metabolic model

Genome scale reconstruction of *S. cerevisiae *reported by Förster *et al*. [29] was used as stoichiometric model of yeast metabolism. All simulations were performed for aerobic glucose-limited conditions. The glucose uptake rate was fixed to 3 mmoles/gDW/hour while the maximum oxygen uptake rate was set to 9 mmoles/gDW/hour [30].

### FBA and MOMA

FBA simulations were performed using the GNU linear programming kit , while MOMA calculations were performed by using an Object oriented quadratic programming package [31].

### Genetic algorithm

The genetic algorithm was implemented as a C++ program using the GAlib package .

#### Note 1: Reported growth rates for mutants

As discussed in the main text, FBA (and other steady state models) can not simulate "rate" without specification of the specific substrate uptake rates (substrate uptake per unit biomass per unit time). Consequently the reported growth rates for the mutants should be more correctly interpreted as biomass yields.

#### Note 2: Maximum theoretical yield of succinate

The maximum theoretical yield of succinic acid reported in this study is calculated using FBA, whereas external H^+ ^was balanced. In case where H^+ ^is regarded as unbalanced (or external) metabolite, maximum yield is 0.98 g/g glucose. This difference is very high and hence can result in big differences in the predictions reported. However, the choice is not trivial since the exact mechanism by which succinic acid is transported out of cell is unknown. Moreover, in case where H^+ ^is not balanced, certain contradictions with the experimental observations were found under anaerobic conditions. For this reason we chose to use a conservative estimate for the maximum theoretical yield. We also note that the theoretical yields were calculated with the constraints for maintenance cost, and no CO_2 _uptake. Thus the reported yields are slightly lower than the stoichiometric yields (1.124 g/g glucose in case of succinate).

#### Note 3: Data availability

The flux distributions, model reactions and other data related to this article can be obtained for non-profit research purposes by contacting the corresponding author (JN).

## Authors' contributions

KRP, IR and JN designed the research. KRP performed the simulations. KRP, IR, JF and JN analyzed the results. All authors read and approved the final manuscript.

## Supplementary Material

Additional File 1 All Supplementary figures and tables. Click here for file
