# Supplementary material for: Evolutionary programming as a platform for in silico metabolic engineering
Source: BMC Bioinformatics. 2005 Dec 23;6:308. doi: 10.1186/1471-2105-6-308 (PMC1327682; doi:10.1186/1471-2105-6-308)
Supplement: Additional File 1 — All Supplementary figures and tables. [file 1471-2105-6-308-S1.pdf]

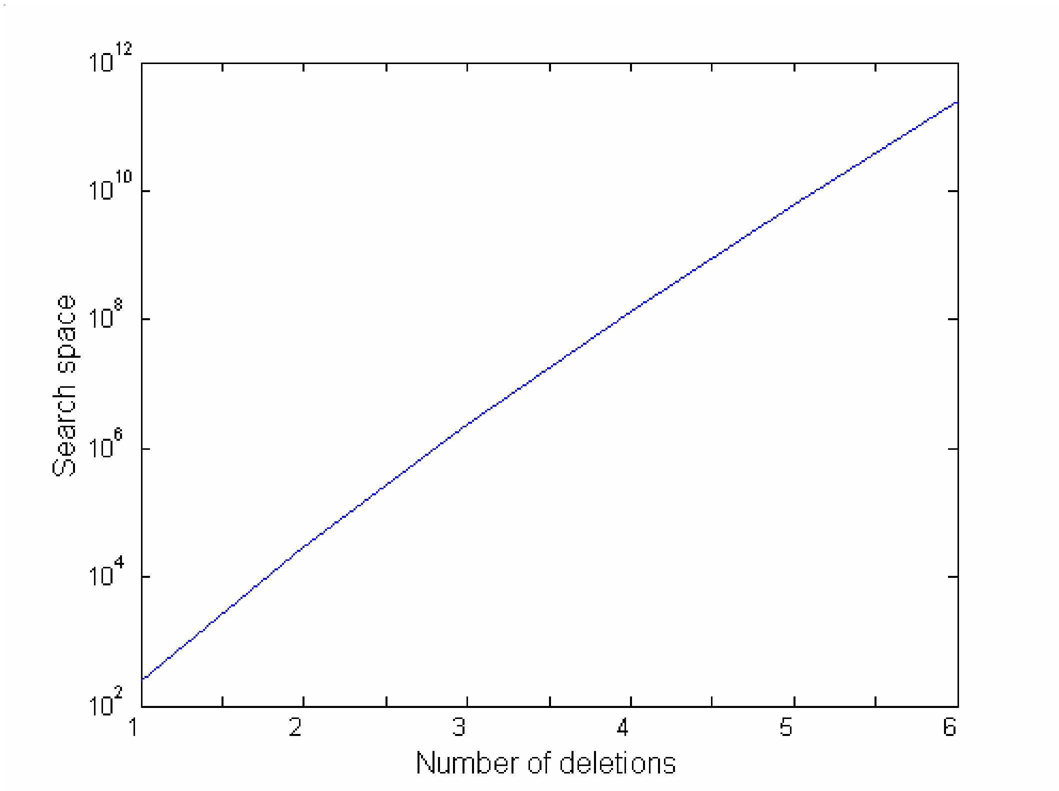

Supplementary Figure 1 - Increase in size of search space (number of possible combinations for given number of deletions) for a reaction-deletion problem with 240 reactions.

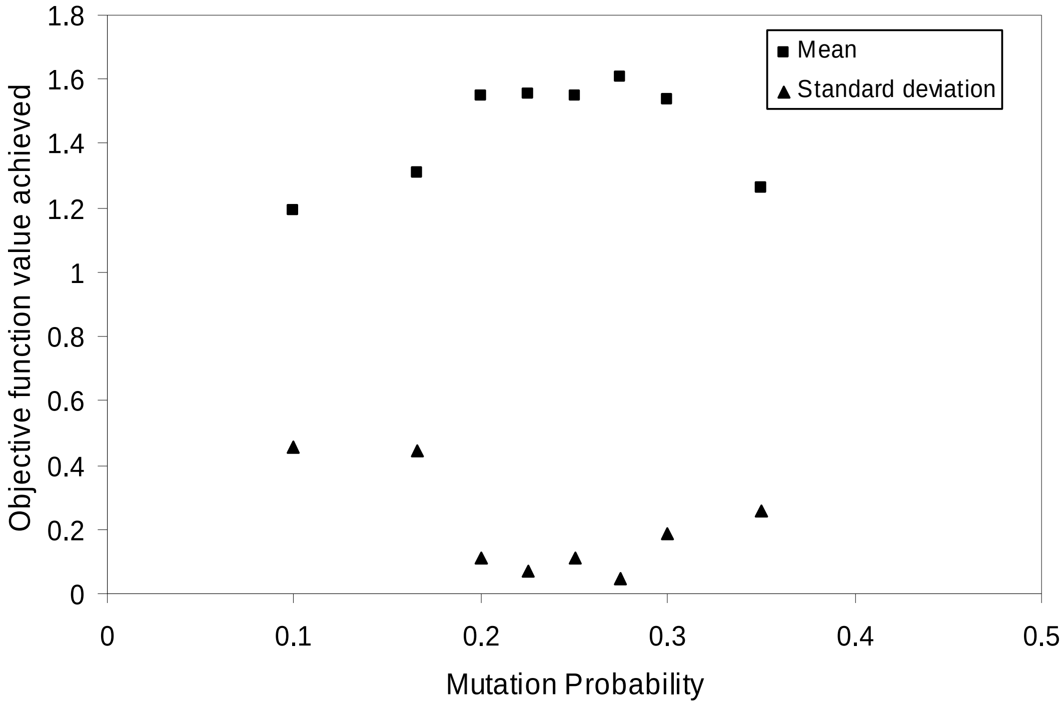

Supplementary Figure 2 - Effect of mutation probability on performance of OptGene. Mean and standard deviation for Succinic acid yield for best individual after fixed number of generations are plotted (arbitrary units).

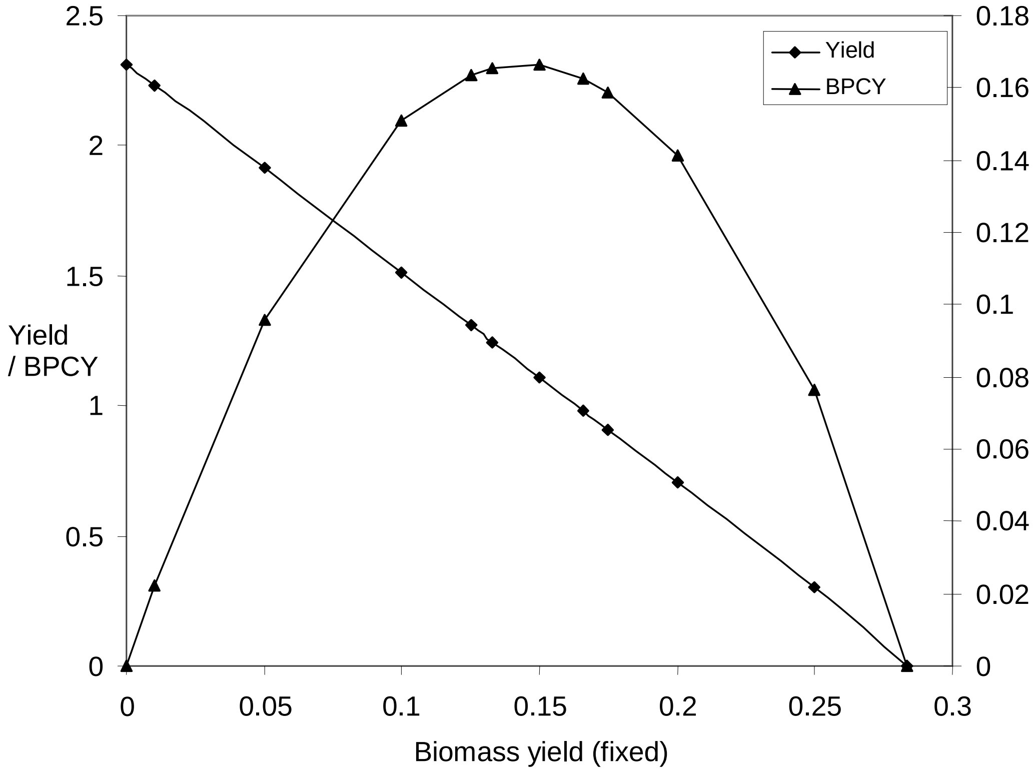

Supplementary Figure 3 - Maximum yield and Biomass Product Coupled Yield of succinate at fixed biomass yield and glucose uptake rate (arbitrary units).

Abbreviation	Metabolite
ACCOAcyt	cytosolic Acetyl Coenzyme-A
ACCOAmit	mitochondrial Acetyl Coenzyme-A
ACA	Acetaldehyde
ACE	Acetate
ACEX	Acetate (extracellular)
AKG	2-Oxoglutarate
CIT	Citrate
CO2	CO2
DHAP	Dihydroxyacetone phosphate
ETH	Ethanol
E4P	D-Erythrose 4-phosphate
F6P	D-Fructose 6-phosphate
F16P	D-Fructose 1,6-bisphosphate
FUM	Fumarate
GA3P	D-Glyceraldehyde 3-phosphate
G6P	D-Glucose 6-phosphate
G15L	D-Glucono-1,5-lactone 6-phosphate
GLOX	Glyoxylate
GLC	Glucose
GP	sn-Glycerol 3-phosphate
ICI	Isocitrate
MAL	Malate
OAA	Oxaloacetate
P13G	3-Phospho-D-glyceroyl phosphate
P2G	2-Phospho-D-glycerate
P6G	6-Phospho-D-gluconate
P3G	3-Phospho-D-glycerate
PEP	Phosphoenolpyruvate
PYR	Pyruvate
R5P	D-Ribose 5-phosphate
RU5P	D-Ribulose 5-phosphate
SUC	Succinate
SUCCOA	Succinyl-CoA
S7P	Sedoheptulose 7-phosphate
X5P	D-Xylose-5-phosphate
ADP	ADP
ATP	ATP
NADPcyt	cytosolic NADP+
NADHcyt	cytosolic NADH
NADcyt	cytosolic NAD+
NADPHcyt	cytosolic NADPH
NADmit	mitochondrial NAD+
NADHmit	mitochondrial NADH
NADPmit	mitochondrial NAD+
NADPHmit	mitochondrial NADPH
FAD	FAD++
FADH2	FADH2
SER	Serine
THR	Threonine
ASP	Aspartate
GLUT	Glutamate

Supplementary Table 1 – Metabolite abbreviations used in the Figure 3 of main text.

Objective function	Number of deletions	Suggested deletions[1]	Objective function value[2]	%Maximum Growth
Glycerol yield	6	*FBA1, TDH1, GDH3, YDR111C, PRO2, MTD1*	0.51	1.2
	3	*FBA1, TDH1, RIP1*	0.49	13
Glycerol Biomass Product Coupled Yield	6	FBA1, PDA1, OSM1, PDC6, GCV1, GAP1	41.12	46.24
	3	*FBA1, TDH1, PDA1*	27.48	20.26

Supplementary Table 2 - Different deletion strategies suggested by OptGene algorithm for improving glycerol yield and Biomass Product Coupled Yield.

[1] Only few of the suggested strategies, with high objective function values are shown. OptGene found many strategies with different, but high objective function values. This tendency can be controlled by varying GA parameters.

[2] Units are: Yield in gram (gram glucose)$^{-1}$,

Biomass Product Coupled Yield in milli-gram (gram-glucose.hour)$^{-1}$

L-Glutamate, Glucose, 2-Oxoglutarate, Glycerol, L-Alanine, L-Arginine, L-Asparagine, L-Aspartate, L-Cysteine, Glycine, L-Glutamine, L-Histidine, L-Isoleucine, L-Leucine, L-Lysine, L-Methionine, L-Ornithine, L-Phenylalanine, L-Proline, L-Serine, L-Threonine, L-Tryptophan, L-Tyrosine, L-Valine, Guanine, HYXN, Xanthine, Acetate, Formate, Ethanol, Succinate, Urea, Orthophosphate, Citrate, Fumarate, (R)-Pantothenate, CO2, Acetaldehyde, Adenosine 3',5'-bisphosphate, dTTP, Thymine, D-Glucosamine 6-phosphate, 8-Amino-7-oxononanoate, Malate

Supplementary Table 3 – List of metabolites that were allowed to be secreted out during OptGene simulations.

Number of deletions	Total search space	OptGene search space (Yield)	OptGene search space (BPCY)
3	2275280	115485	184677
4	1.35E+08	42712	452000
5	6.36E+09	103875	51750

Supplementary Table 4 – Computational performance of OptGene as compared with the exhaustive search. Since CPU time will be dependent on the machine type and linear programming solver used, we have reported “search space” (or number of objective function evaluations) for Succinic acid case study. However, we note that the CPU time will be proportional to the number of objective function evaluations, independent of the machine type and solver. The fraction of independent runs converging to the best solution was taken into account while estimating the number of objective function evaluations for OptGene.
